# Salvage versus amputation: Utility of mangled extremity severity score in severely injured lower limbs

**DOI:** 10.4103/0019-5413.33679

**Published:** 2007

**Authors:** M Kiran Kumar, CM Badole, KR Patond

**Affiliations:** Department of Orthopedics, Narayana Medical College, Chinthareddypalem, Nellore - 524 002, (AP), India; * Department of Orthopedics, MGIMS, Sewagram - 442 102, (Wardha), India

**Keywords:** Mangled lower extremity, Mangled extremity severity score, salvage versus amputation

## Abstract

**Background:**

The purpose of the present study was to evaluate the clinical utility of Mangled extremity severity score (MESS) in severely injured lower limbs.

**Materials and Methods:**

Retrospectively 25 and prospectively 36 lower limbs in 58 patients with high-energy injuries were evaluated with the use of MESS, to assist in the decision-making process for the care of patients with such injuries. Difference between the mean MESS scores for amputated and salvaged limbs was analyzed.

**Results:**

In the retrospective study 4.65 (4.65 ± 1.32) was the mean score for the salvaged limbs and 8.80 (8.8 ± 1.4) for the amputated limbs. In the prospective study 4.53 (4.53 ± 2.44) was the mean score for the salvaged limbs and 8.83 (8.83 ± 2.34) for the amputated limbs. There was a significant difference in the mean scores for salvaged and amputated limbs. Retrospective 21 (84%) and prospective 29 (80.5%) limbs remained in the salvage pathway six months after the injury.

**Conclusion:**

MESS could predict amputation of severely injured lower limbs, having score of equal or more than 7 with 91% sensitivity and 98% specificity. There was a significant difference in the mean MESS scores in the prospective study (n=36), 4.53 (4.53 ± 2.44) in thirty salvaged limbs (83.33%) and 8.83 (8.83 ± 2.34) in six amputated limbs (16.66%) with a *P*-value 0.002 (*P*-value < 0.01). Similarly there was a significant difference in the mean MESS score in the retrospective study (n=25), 4.65 (4.65 ± 1.32) in twenty salvaged limbs (80%) and 8.80 (8.8 ± 1.4) in five amputated limbs (20%) with a *P*-value 0.00005 (*P*-value < 0.01). MESS is a simple and relatively easy and readily available scoring system which can help the surgeon to decide the fate of the lower extremity with a high-energy injury.

Massive lower extremity trauma, in particular open tibial fractures with associated vascular injuries, presents immediate and complex decision-making challenges between a limb salvage attempt and primary amputation. The management of massive lower extremity trauma is a subject of considerable interest and controversy. While the evolution of sophisticated microsurgical reconstruction technique has created the possibility of successful limb salvage in even the most extreme cases, it has become painfully obvious that the technical possibilities are double-edged swords.^1^

Hansen^2^ in analyzing his vast personal experience with managing open fractures, noted that protracted limb salvage attempts may destroy a person physically, psychologically, socially and financially, with adverse consequences for the entire family as well. In spite of best attempts, the functional results are often worse than an amputation. Thus, enthusiasm for limb salvage techniques must be tempered by a realistic assessment of the results, not just for the injured part but for the patient as a whole.

The aim of limb salvage procedures following severe lower limb trauma is the preservation of a viable and fully functional limb. Unfortunately, while limb preservation is frequently possible, the salvaged limb may have significant functional deficits and may have ultimately required secondary ablation. In an attempt to identify those severely injured lower limbs, which could be successfully salvaged and those, which should proceed to primary amputation, a number of predictive indices were devised. These included the Mangled extremity severity index (MESI), the Mangled extremity severity score (MESS), predictive salvage index (PSI) and the limb salvage index (LSI).^3^

## MATERIALS AND METHODS

This study was carried out from January 1998 to June 2004. The study group comprised all the patients of either sex and any age, who had presented in emergency. The study includes a total of 58 patients with 61 limbs. The details of the retrospective (n=25) cases were obtained from the medical records. Cases were selected as per the following inclusion criteria:

1. Mangled lower extremity
2. Gustilo Type-IIIA femur and tibial fractures with a hospital stay of more than four days, severe muscle damage, associated nerve injury and major blood loss or bone injury; associated with a fibular fracture and displacement of more than 50% and comminuted and segmental fracture
3. Gustilo Type-IIIB femur and tibial fractures
4. Gustilo Type-IIIC femur and tibial fractures
5. Vascular injuries of lower limb except the foot, including dislocations of the knee, ankle, closed tibial or femoral fractures and penetrating wounds with vascular injury noted on color Doppler or at the time of surgery
6. Gustilo Type-III open pilon fractures

Cases that are excluded from this study were

1. The injured limbs that were traumatic, near-amputation with only a small bridge of tissue connecting the distal extremity, thus were not reconstructable.
2. Severely injured limbs with an unreconstructable foot,
3. Patients with traumatic limb avulsions, isolated foot or digit injury and
4. Patients who expired in less than one week from admission.

On admission to emergency ward all resuscitative measures according to the advanced trauma life support (ATLS) protocol were followed. Once the general condition of the patient was stabilized, a detailed case history was recorded with particular importance to mode of injury, treatment taken if any, interval between injury and admission and associated medical or surgical illnesses if any. Radiographs of the mangled extremity were taken. For all the mangled extremities pulse oximeter reading was noted and monitored till improvement of vascularity. Color Doppler of mangled extremity was carried out whenever peripheral pulses were absent and perfusion was in doubt. Patients were shifted to the operation room and initial management of the mangled extremity was started in the form of thorough irrigation with copious normal saline, meticulous debridement, pressure bandage, antibiotics and fracture stabilization with external fixator. MESS was done at the time of admission or on the operation table, according to Table 1.^4^

**Table 1:** Mangled extremity severity score^4^

Type	Characteristics	Injuries	Points
Skeletal/soft-tissue Group			
1	Low-energy	- Stab wounds, simple closed fractures, small-caliber gunshot wounds	1
2	Medium-energy	- Open or multiple-level fractures, dislocations, moderate crush injuries	2
3	High-energy	- Shotgun blast (close range) high-velocity gunshot wounds, crush injury	3
4	Very high-energy	- Above + gross contamination, soft tissue avulsion.	4
Shock group			
1	Normotensive hemodynamics	- BP stable in field and in operation theatre	0
2	Transiently hypotensive	- BP unstable in field but responsive to intravenous fluids	1
3	Prolonged hypotension	- Systolic BP less than 90mmHg in field and responsive to intravenous fluids only in operation theatre	2
Ischemia group			
1	None	- A pulsatile limb without signs of ischemia	0*
2	Mild	- Pulse reduced or absent but perfusion normal	1*
3	Moderate	- Pulseless; parasthesia, diminished capillary refill	2*
4	Advanced	- Pulseless, cool, paralyzed and numb without capillary refill	3*
Age group			
1	< 30 years		0
2	> 30 - <50 years		1
3	> 50 years		2

* Points × 2 if ischemia time exceeds six hours,

BP - Blood pressure

Vascular repair, if indicated, and primary fracture alignment and stabilization were carried out. Serial debridements were carried out every two to four days when required. The second look debridement under anesthesia was undertaken 48 to 72 hrs following the injury. Serial wound cultures were done and appropriate antibiotics were given. Adequate physiotherapy was carried out depending upon the circumstances. This salvage protocol was abandoned if the general condition of the patient deteriorated or once the severe infection of injured limb was observed or renal failure set in making amputation inevitable. Gradual delayed primary closure / split-thickness skin grafting / myocutaneous flap coverage was undertaken when required. Iliac bone grafting was undertaken in patients with bone loss or lack of healing process at the fracture site. Once adequate soft tissue coverage had been obtained, patient was discharged and followed up at regular intervals of two weeks for progression of fracture healing. External fixator was replaced with a cast when there was no sign of infection, adequate soft tissue coverage was obtained and the fracture healing was progressing satisfactorily. External fixator or cast was removed once the fracture was soundly united and adequate physiotherapy was advised.

In the retrospective study, all the attempted salvage patients were followed up for a period of two and a half years. Maximum follow-up in the study period was done at the end of six and a half years, with minimum follow-up done at the end of two and half years. Three patients had not reported for follow-up and two patients had died before complete follow-up. In the prospective study all the attempted salvage patients were followed up for a minimum period of six months. A successful salvage limb was defined as an extremity that remained in the limb salvage and reconstruction pathway six months after injury. Six months were selected as the end point because patients who had amputation after that time would be most likely to have had major complications or intolerance to additional reconstruction efforts or both.^5^ Maximum period of follow-up in the study period was 28 months; minimum follow-up period was two months. Average duration of follow-up was six months. No patient died within the follow-up period.

## RESULTS

The present study comprised 58 patients with 61 injured lower limbs. The retrospective study group comprised 24 patients with 25 injured limbs and the prospective study group comprised 34 patients with 36 injured limbs. Most of the patients were males (n=45, 84.6%) and the mean age of the patients was 34.5 (75-8) years. Right lower limb was commonly injured (n=35, 57%). Crush injury of leg with fracture of tibia and fibula was observed in 80% of injured limbs. The most common mechanism of injury was high-energy trauma. Road traffic accidents accounted for 70.8% of patients.

The mean hospitalization for primary amputation was 18.2 (7-25) days and for delayed amputation limbs was 35.5 (14-60) days and for salvaged limbs was 44.5 (13-132) days. There were 15 (24.5%) of Gustilo Type IIIA limbs, 25 (41%) of Type IIIB and 21 (34.5%) of Type IIIC fractures observed. All the injured limbs with MESS score of equal or more than 7 were of Gustilo Type IIIC.

In the retrospective study, all the five (20%) injured limbs with a MESS score of equal or more than 7 were amputated (mean score 8.8 with range of 10-8) and the remaining 20 (80%) injured limbs with a MESS score of less than 7 were salvaged (mean score 4.65 with range of (4-6)); suggesting a significant difference in the mean scores. In the retrospective study of 25 injured limbs, four limbs (20%) had primary amputation and one limb (5%) had delayed amputation.

In the prospective study, out of six (16.66%) injured limbs with a MESS score of equal or more than 7, five limbs (13.88%) were amputated and one limb (2.78%) was salvaged. Out of the remaining 30 injured limbs (83.44%) with a MESS score of less than 7, 29 limbs (80.55%) were successfully salvaged and one limb (2.78%) was amputated. The mean score for salvaged limbs was 4.5(3-7) and for amputated limbs was 8.83(6-12), suggesting a significant difference in the mean scores. In the prospective study of 36 injured limbs, three limbs (8.33%) had primary amputation and three limbs (8.33%) had delayed amputation. In the prospective study, maximum period of follow-up was 28 months and minimum period was two months. In the retrospective study, maximum period of follow-up was done at the end of six and half years and minimum period of follow up was done at the end of two and half years.

Out of a total of 61 injured lower limbs, 11 limbs (18.03%) were amputated, 43(70.5%) salvaged limbs had good function, four salvaged limbs (6.56%) had poor function and three attempted salvaged limbs (4.9%) were lost to follow-up. MESS could predict amputation of severely injured lower limbs, having score of equal or more than 7 with 91% sensitivity and 98% specificity.

## DISCUSSION

The management of severe lower limb injury remains one of the most controversial subjects in Orthopedic surgery. Advances in surgical technology of fracture fixation, infrapopliteal vascular reconstruction and free micro vascular tissue transfer now permit limb salvage in the majority of lower limb trauma cases. Unfortunately, while most attempts of limb salvage are successful, many are not. Failed attempts at limb salvage result in prolonged hospitalization including multiple surgical procedures, pain and psychological trauma, as well as economic hardship to the patient. Frequently, overzealous attempts at limb salvage with prolonged unsuccessful attempts at rehabilitation result in a functionally useless limb, chronic disability and pain and may be followed later by delayed amputation. In contrast, correct application of surgical salvage technique may successfully rescue a limb, which might otherwise have been amputated. The ideal situation is one which allows identification of those patients who will benefit from early and aggressive attempts at limb salvage and those for whom primary amputation is the correct choice.^3^

An attempt to quantify the severity of the trauma and to establish numerical guidelines for the decision to amputate or salvage the limb has been proposed by many authors. These include the MESS, the PSI, the LSI, the nerve injury, ischemia, soft tissue injury, skeletal injury and age of the patient (NISSA) score and the Hanover fracture scale-97 (HFS-97).^5^

Johansen *et al.,*^4^ proposed the MESS based on four clinical criteria: skeletal/soft tissue injury, limb ischemia, shock and age [Table 1]. A point system was developed to grade the severity of each of the four criteria. The MESS was based on retrospective review of 26 limbs. They also reported a prospective trial validating by index with 26 patients at a separate trauma center. They concluded that a MESS score of less than 7 predicted salvage with 100% accuracy and a MESS score of equal or more than 7 predicted amputation with 100% accuracy.

Lange *et al.,*^6^ proposed a decision-making protocol based on absolute and relative indications. The occurrence of just one of two absolute indications (complete posterior tibial nerve disruption in adults; crush injuries with longer than six hours of warm ischemia time) warrants primary amputation, while at least two of three relative indications (serious associated polytrauma, severe ipsilateral foot trauma or projected long course to full recovery) must be present to reach that decision.

Russell *et al.,*^7^ proposed a Limb Salvage Index (LSI) scoring system, based on the analysis of 70 lower extremity injuries involving multiple systems. LSI was formulated based on the degree of injury to the arterial, nerve, bone, muscle, skin, venous and warm ischemia time. LSI score of less than 6 predicts successful limb salvage whereas LSI score of 6 or more than six predicts amputations.

McNamara *et al.,*^8^ introduced the nerve injury, ischemia, soft tissue injury, skeletal injury, shock and age of patient (NISSSA) score, to address accurate decision-making of salvage versus amputation in severely injured lower extremities.

Slauterbeck *et al.,*^9^ conducted a study on 37 patients having 43 mangled upper extremities. All nine extremity injuries with a MESS score of equal to or more than 7 were amputated and 34 with a MESS of less than 7 were salvaged. They concluded that the MESS was an early and accurate predictor for identifying the extremities that may be treated by amputation.

O'Sullivan *et al.,*^3^ retrospectively applied MESS and LSI to 54 extremities in 50 patients of Gustilo Type III B and Type III C tibial fractures and observed that MESS was more accurate than LSI in predicting limb salvage. A MESS score of more than 7 offered a greater relative risk of amputation than an LSI score of more than 6. However, when the limbs which required delayed amputation were analyzed, the LSI was slightly more accurate in predicting amputation.

Farris *et al.,*^10^ tested the ability of MESS to predict the outcome of amputation in 119 patients with 122 blunt injuries to the lower limb associated with arterial injuries. They reported that MESS had a positive predictive value of 71%, a negative predictive value of 84% and an overall accuracy of prediction of 75%. They concluded that MESS is not sufficiently precise to allow the decision regarding amputation to be made at the initial operation.

In India, Sharma *et al.,*^11^ prospectively applied MESS to 50 patients with 56 mangled upper and lower extremities and after a follow-up of six months, found that MESS had high specificity and high sensitivity, suggesting that MESS score of equal to or more than 7 had 100% predictive value of amputation. Similar results are also found by Lin *et al.*^12^ in their retrospective study on 34 patients with 36 mangled lower extremities with Gustilo Type III C.. Results of both these studies suggest that many limbs with MESS score of equal to or more than 7 may be salvaged. The high sensitivity suggests that almost all limbs requiring an amputation will have MESS equal to or more than seven. Comparison of mean MESS score was made with various studies [Table 2].

**Table 2:** Comparison of mean Mangled extremity severity score in the other studies

Study (Year)	Mean in salvaged limbs	Mean in amputated limbs
*Johansen et al.*,^4^		
Retrospective study	4.88	9.11
Prospective study	4.00	8.83
O'Sullivan^3^		
Retrospective study	3.80	7.70
Pimple *et al.*,^5^		
Retrospective study	6.94	9.40
Sharma *et al.*,^11^		
Prospective study	4.70	8.60
Present study		
Retrospective	4.65	8.80
Prospective	4.50	8.83

The clinical utility of MESS has been extensively evaluated with varying results. There was a significant difference in the mean MESS scores in the prospective study, 4.53 (4.53 ± 2.44) in 30 salvaged limbs (83.33%) and 8.83 (8.83 ± 2.34) in six amputated limbs (16.66%) with a *P*-value 0.002 (*P*-value < 0.01). Similarly there was a significant difference in the mean MESS score in the retrospective study, 4.65(4.65 ± 1.32) in 20 salvaged limbs (80%) and 8.80(8.8 ± 1.4) in five amputated limbs (20%) with a *P*-value 0.00005 (*P*-value < 0.01). MESS in our study had a high specificity (98%) and a high sensitivity (91%), suggesting the few limbs with MESS equal to or more than 7 may be salvaged. The high sensitivity suggests that almost all limbs requiring an amputation will have MESS equal to or more than 7. Results of the present study are consistent with the western and Indian studies [Table 3]. Boss *et al.*^13^ prospectively evaluated 556 high-energy lower extremity injuries with the use of five injury severity scoring systems (MESS, LSI, PSI, NISSSA and HFS) for lower extremity trauma designed to assist in the decision-making process for the care of the patients with such injuries. The sensitivity, specificity and area under the receiver operating characteristic curve was calculated for the MESS, LSI, PSI, NISSSA and HFS for ischemic and non-ischemic limbs. The analysis did not validate the clinical utility of any of the scoring systems.

**Table 3:** Comparison of results with other studies

Study (year)	Patients (limbs)	Follow-up period	Result
Johansen *et al.*^4^			
Retrospective	26 (26)	1 year	High specificity and High sensitivity
Prospective	26 (26)	1 year	
O'Sullivan *et al.*,^3^			
Retrospective	51 (54)	10 years	High specificity and High sensitivity
Lin *et al.*,^12^			
Retrospective	34 (36)	3 years	Low specificity and High sensitivity
Boss *et al.*,^13^			
Prospective	556 (572)	3 years	High specificity and Low sensitivity
Sharma *et al.*,^11^			
Prospective	50 (56)	3 years	High specificity and High sensitivity
Pimple *et al.*,^5^			
Retrospective	26 (26)	2 years	Low specificity and High sensitivity
Present study			
Retrospective	24 (25)	4 years	High specificity and High sensitivity
Prospective	34 (36)	2.5 years	

We caution to keep realistic expectations regarding the ultimate functional result. Both the patient and surgeon must anticipate multiple subsequent operative procedures, a long-term commitment to rehabilitation and a high probability of significant sequelae and functional limitations as an end result in these serious high injuries.
